# Harnessing the Neurobiology of Resilience to Protect the Mental Well-Being of Healthcare Workers During the COVID-19 Pandemic

**DOI:** 10.3389/fpsyg.2021.621853

**Published:** 2021-03-18

**Authors:** Ravi Philip Rajkumar

**Affiliations:** Department of Psychiatry, Jawaharlal Institute of Postgraduate Medical Education and Research, Pondicherry, India

**Keywords:** resilience, stress, neurobiology, neuroendocrinology, neuropeptides, cortisol, coronavirus disease 2019, epigenetics

## Abstract

Healthcare workers are at a high risk of psychological morbidity in the face of the COVID-19 pandemic. However, there is significant variability in the impact of this crisis on individual healthcare workers, which can be best explained through an appreciation of the construct of resilience. Broadly speaking, resilience refers to the ability to successfully adapt to stressful or traumatic events, and thus plays a key role in determining mental health outcomes following exposure to such events. A proper understanding of resilience is vital in enabling a shift from a reactive to a proactive approach for protecting and promoting the mental well-being of healthcare workers. Research in the past decade has identified six areas that provide promising leads in understanding the biological basis of individual variations in resilience. These are: (1) the key role played by the monoamines noradrenaline and serotonin, (2) the centrality of the hypothalamic-pituitary-adrenal axis in influencing stress vulnerability and resilience, (3) the intimate links between the immune system and stress sensitivity, (4) the role of epigenetic modulation of gene expression in influencing the stress response, (5) the role played by certain neuropeptides as a natural “brake” mechanism in the face of stress, and (6) the neurobiological mechanisms by which environmental factors, such as exercise, diet, and social support, influence resilience to subsequent life events. Though much of this research is still in its early stages, it has already provided valuable information on which strategies – including dietary changes, lifestyle modification, environmental modification, psychosocial interventions, and even pharmacological treatments – may prove to be useful in fostering resilience in individuals and groups. This paper examines the above evidence more closely, with a specific focus on the challenges faced by healthcare workers during the COVID-19 pandemic, and provides suggestions regarding how it may be translated into real-world interventions, as well as how the more tentative hypotheses advanced in this field may be tested during this critical period.

## Introduction

### A Cautionary Tale

The COVID-19 pandemic has taken a significant toll on the psychological well-being of healthcare workers, and that this impact remains substantial even in those who are not directly involved in caring for patients with this disease (da Silva and Neto, 2020). Based on experience from earlier outbreaks of similar severity and smaller scope (Maunder et al., 2008; Lee et al., 2018), this phenomenon was predicted well in advance, and in many cases, plans and services were developed to minimize the traumatic impact of COVID-19 on healthcare workers as the pandemic began to evolve and take on a truly global scope. One of the earliest reports of such an attempt, published in February, came from the Second Xiangya Hospital of Central South University, China, which was at the center of the initial COVID-19 outbreak (Chen et al., 2020a). The importance of this report, despite its anecdotal nature, was that it highlighted the limitations of the “conventional” approach to such problems. At this hospital, which was handling a large number of COVID-19 cases, a three-pronged approach was devised by a team of experts, which consisted of (i) a psychological intervention team which provided online courses to address common mental health problems, (ii) a dedicated hotline, and (iii) group psychological activities to minimize stress. Such an approach was in line with recommendations from the existing literature. However, the majority of staff were unwilling to participate in these activities, and many of them refused assistance from the team despite showing obvious signs of distress.

Interviews with the staff revealed that this program did not address their real-world concerns, which included separation from their families, difficulties in handling the anxieties of patients, worries regarding shortages of food, protective equipment, and other essential supplies, and a lack of time for sleep or leisure. This feedback led to an overhaul of the entire program, which now included (i) ensuring the availability of food and essential supplies, (ii) training staff to handle patients’ concerns, (iii) provision of a rest area and leisure activities, and (iv) periodic visits by a counselor; on the other hand, there was a reduced emphasis on the exclusively psychological or counseling-based interventions which formed part of the initial plan. This approach led to greater satisfaction and a reduction in perceived stress among nursing and other staff.

What can we learn from the initial failure and later success of such programs? At a surface level, they highlight the need to listen to healthcare workers’ actual concerns when designing interventions to improve their psychological well-being. However, a deeper insight into such occurrences can be obtained by a careful study of contemporary research into resilience, the multiple and interconnected biological mechanisms that underlie it, and the way in which resilience can be fostered by methods such as exercise, socialization, and environmental modification. The purpose of this paper is to provide an overview of this research, with a particular focus on how it might apply to the psychological health of healthcare workers in the context of the COVID-19 pandemic, and outline suggestions for how this knowledge can be translated into effective strategies for the prevention and management of psychological distress in this population.

### Reactive and Proactive Approaches to Psychological Health in Healthcare Workers During the COVID-19 Pandemic

In a meta-analytic review of 13 observational studies, Pappa et al. (2020) have estimated that 23.2% of healthcare workers experience significant symptoms of anxiety in the context of the COVID-19 pandemic; 22.8% report significant depressive symptoms; and 38.9% screen positive for insomnia. Similar results were obtained in a meta-analysis of eight studies dealing exclusively with frontline healthcare workers (da Silva and Neto, 2020). Many of these observational studies have concluded with recommendations for the monitoring and treatment of healthcare workers with such symptoms (Huang and Zhao, 2020; Zhang et al., 2020b); however, only one paper pointed out the potential benefits of a preventive approach (Li et al., 2020). While it is essential that healthcare workers with emergent symptoms of psychological distress are identified and treated early, there are advantages to supplementing this conventional model with an approach based on enhancing the abilities of asymptomatic healthcare workers to cope with stress – in other words, with a resilience-based approach. Such a proactive approach will continue to gain importance as the COVID-19 pandemic continues to evolve, and even after it begins to abate, as large numbers of healthcare workers will remain exposed to stress, socioeconomic difficulties and ethical challenges over a prolonged period of time (Vinkers et al., 2020). The advantages of a proactive approach informed by an understanding of resilience include not only the prevention and mitigation of psychological distress, but improved functioning and an enhanced capacity to handle challenging or unpredictable situations in patient care, particularly in the setting of a scarcity of resources (Rosen et al., 2020; Vinkers et al., 2020). In addition, if successful, such an approach would reduce the burden faced by conventional mental health care services, and permit them to provide optimal care to those healthcare workers with more severe symptoms and greater treatment needs (Freeman, 2020).

### Understanding Resilience: Psychological and Neuroscience-Based Approaches

Resilience can be defined as “the ability to adapt successfully in the face of stress and adversity” (Wu et al., 2013). In other words, it refers to the capacity to maintain a normal or near-normal level of functioning, even when exposed to a stressful or traumatic event. It is a common-sense observation that, even after exposure to a traumatic event such as a natural or man-made disaster, not all individuals develop symptoms of psychological distress. Moreover, those who do so exhibit varying levels of such symptoms, with severe sequelae being the exception rather than the rule (Rutter, 2012). Resilience is best understood as a continuous, dynamic concept, and not an all-or-none phenomenon, which aims to capture inter-individual variations in biological, psychological, and behavioral responses and outcomes following a stressful event (Zovkic et al., 2013). From a psychological point of view, resilience can be studied in terms of constructs such as self-efficacy, optimism, positive emotions, and cognitive appraisal (Feder et al., 2019) and operationalized in terms of absent or low levels of mental health problems and sustained normal functioning during times of adversity. Some researchers have identified two components to resilience – adversity and positive adaptation – but others have argued for more complex models, particularly on the basis of longitudinal studies (Cosco et al., 2017).

From a neuroscientific perspective, resilience can be defined and studied in terms of changes at the genetic, biochemical, cellular, anatomical, and physiological levels that correlate with responses to adversity, threat, or trauma (Cathomas et al., 2019; Feder et al., 2019; Gururajan et al., 2019). For example, candidate gene and genome-wide analyses have identified genetic factors that are associated with individual responses to stressful events (Stein et al., 2019; Notaras and van den Buuse, 2020); neurochemical studies have identified changes in specific neurotransmitters, such as monoamines and neuropeptides, which correlate with varying responses to stress (Averill et al., 2018); and neuroimaging studies have investigated structural and functional changes in particular brain regions that are related to stress vulnerability (Hanson et al., 2019). A useful model that bridges the conceptual gap between neuroscience and observed responses is the affiliative neuroscience approach outlined by Feldman (2018). From this perspective, which integrates biology and behavior, resilience is viewed in terms of three aspects: *plasticity*, which is the ability of living tissue – in this case, neural tissue – to adapt to changes; *sociality*, which refers to the protective and stress-buffering role of social behaviors and relationships, and *meaning*, which is specific to humans and involves finding significance and strength in the face of suffering and also covers such constructs as spirituality and altruism. This model will be used in this paper when outlining possible links between research findings and the actual needs and experiences of healthcare workers.

### The Need for a Biologically Informed, Resilience-Based Approach to Mental Health, Particularly in Healthcare Workers

In recent times, a growing awareness of the limitations of contemporary models of mental health and illness has led some researchers to critically examine the value of a resilience-based approach to these subjects. Such an approach has already begun to yield fruit in the study of psychiatric disorders such as depression (Elisei et al., 2013; Richter-Levin and Xu, 2018) and post-traumatic stress disorder (PTSD; Yehuda et al., 2016; Olff et al., 2019; Rakesh et al., 2019). Similarly, researchers in the field of child development are beginning to unravel the way in which genes, brain regions, and specific neurotransmitters influence the response of a child’s brain to maltreatment or neglect. This raises the encouraging possibility of using this knowledge to promote resilience in children who have experienced deprivation (Ioannidis et al., 2020).

Such work is of direct relevance to healthcare workers, particularly during the current pandemic. Due to the specific nature of their work and the multiple stressors it may entail, these personnel are at an elevated risk of adverse mental health outcomes, and have been identified as a population that would benefit from resilience-enhancing interventions well before the COVID-19 pandemic. Available evidence suggests that certain “resilience training” programs, based on the mindfulness or cognitive-behavioral models, may have a short-term beneficial effect on perceived stress and depressive symptoms; however, a Cochrane Database systematic review found that the effect sizes for these interventions were small, and the certainty that could be attributed to any positive results was low (Kunzler et al., 2020). Moreover, a neurobiological evaluation of one such “stress management training” program found that it did not significantly alter the cortisol response to stress, and even worsened it in some participants, suggesting that such interventions may fail to achieve optimal results because they do not lead to relevant changes at the cellular or neural level (Gloster et al., 2019). In the context of such results, there is a significant need for approaches that adapt the principles of the neurobiology of resilience to the healthcare context (Llinas et al., 2018), a need that takes on a particular urgency as the world prepares itself for a “second surge” of the COVID-19 pandemic (Benham et al., 2020).

### The Focus of the Current Paper

Though hundreds of papers have been published in this field in recent years, for the sake of brevity and clarity, the current paper has chosen to focus on six specific areas. These six domains are:

- The contemporary understanding of monoamine transmitter systems, particularly those involving noradrenaline and serotonin, in modulating stress response and resilience.
- The central role of psychoneuroendocrine mechanisms, particularly those involving the hypothalamic-pituitary-adrenal axis (HPA), as a putative “final common pathway” mediating vulnerability and resilience to stress.
- The key links between the immune system and the stress response, in terms of both risk and resilience.
- The epigenetic regulation of key genes involved in the stress response, and the role of this process in mediating resilience.
- The functions of certain peptide transmitters, such as neuropeptide Y (NPY) and oxytocin, in moderating the effects of stress and acting as a natural “brake” mechanism in this context.
- The neurobiological mechanisms by which environmental factors, such as early life stress, exercise, and social support, influence resilience to subsequent life events.

**Figure 1:**
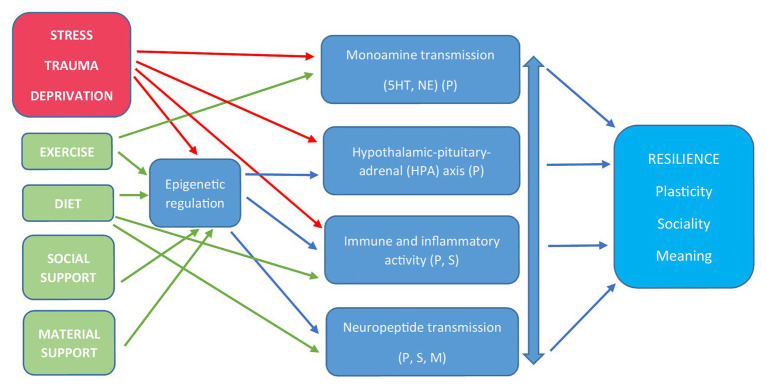
An overview of biological mechanisms underlying stress susceptibility and resilience. Blue arrows indicate regulatory mechanisms and pathways. Red arrows indicate negative effects and green arrows indicate positive effects. Interrelations between the four common molecular mechanisms are reciprocal, as indicated by the double arrow. 5-HT, serotonin; NE, norepinephrine; HPA, hypothalamic-pituitary-adrenal; P, plasticity; S, sociality; M, meaning.

Three factors influenced the decision to focus on these domains. First, they have been identified as foci of particular research interest and activity in recent reviews (Fleshner et al., 2011; Wu et al., 2013; Averill et al., 2018; Feder et al., 2019). Second, they can be easily related to the affiliative neuroscience framework outlined by Feldman (2018) in a more or less hierarchical manner: the first two are more directly related to *plasticity*, while the last four provide a bridge from *plasticity* to *sociality* and *meaning*. Finally, and most importantly, they provide potential or actual targets for intervention that can be tested with relative ease in the current context. It is not the purpose of this article to provide a systematic review of work in this field, but rather to illustrate the potential value of this approach through certain key examples. A broad outline of these mechanisms and the interplay between them is provided in Figure 1.

## Key Research Areas in the Neurobiology of Resilience and Their Application to the COVID-19 Healthcare Worker Scenario

### Monoaminergic Modulation of Stress and Resilience

A consistent body of evidence supports the notion that monoamine transmitters are key modulators of human emotional and behavioral responses to stress. In the context of a prolonged traumatic situation such as the COVID-19 pandemic, the two transmitters of greatest potential significance are noradrenaline, which mediates the “flight or fight” response to stress, and serotonin, which is involved in risk appraisal and in the emotions of sadness and anxiety (Wang et al., 2020b). Variations in genes involved in regulating the function of these two transmitters, such as the catechol-O-methyltransferase (*COMT*) gene, the monoamine oxidase type A (*MAOA*) gene, the tryptophan hydroxylase type II gene (*TPH2*), and the serotonin transporter (*SLC6A4*), have been associated with significant variations in stress response and resilience in both human and animal subjects (Jabbi et al., 2007; Clukay et al., 2019; Gonzalez-Giraldo and Forero, 2020; Strekalova et al., 2020). Research in mice suggests that the noradrenergic system plays a particular role in influencing resilience to chronic stress (Isingrini et al., 2016), and that the activation of a specific serotonin receptor subtype (5HT_4_) reduces both fear-like and depressive-like responses to chronic stress (Chen et al., 2020b). Drugs that putatively influence resilience *via* other receptors, such as ketamine, have also been shown to depend on intact monoamine systems for their effects in preventing stress-induced depression (Brachman et al., 2016; Bowman et al., 2020).

These effects are mediated both by the direct post-synaptic effects of these transmitters, and by their “cross-talk” with other pathways such as the midbrain dopaminergic pathway (Krystal and Neumeister, 2009), peptides such as NPY (Hokfelt et al., 2018; see section Neuropeptides), and, most significantly, the HPA “stress axis” (Ancelin et al., 2017; Prakash et al., 2020; see section The Hypothalamic-Pituitary-Adrenal Axis). Translational evidence has identified neural “final common pathways” for these effects. For example, the noradrenergic locus coeruleus (NOR-LC) system, connecting the brainstem with the amygdala, which modulates the formation and consolidation of memories related to stressful or traumatic events (Haubrich et al., 2020). Similarly, plasticity of midbrain serotonergic neurons has been associated with resilience to stress-induced depressive symptoms (Prakash et al., 2020).

At a higher level of analysis, genetic variations in serotonergic transmission have been associated with varying levels of psychological flexibility (Gloster et al., 2015), which is a key variable influencing coping strategies and stress resilience during the COVID-19 pandemic (Smith et al., 2020). Similarly, heart rate variability (HRV), a cardiac index influenced by noradrenergic transmission, is an important modulator of the neuroendocrine stress axis (Kemp and Quintana, 2013; see section Epigenetic Regulation of the Stress Response) and is itself correlated with psychological flexibility. Direct evidence for a link between these parameters was observed in a study of patients with depression, in which the level of occupancy of norepinephrine transporters by the antidepressant venlafaxine was associated with both improved resilience and increased HRV (Davidson et al., 2005). Studies of both civilians and military personnel have found that elevated levels of norepinephrine are associated with an increase subsequent risk of PTSD, while lower levels predict resilience (Highland et al., 2015). Similarly, a study of nurses working in operating rooms found that elevations in peripheral levels of norepinephrine were significantly associated with the development of post-traumatic disorder (Ke et al., 2020), and that this alteration was also associated with immune dysregulation (see section Immune-Inflammatory Influences on Stress and Resilience).

Taken together, these results suggest that variations in monoaminergic functioning can potentially influence several downstream neuroendocrine, neuroimmune, and neurocognitive processes through alterations in *plasticity* (Levone et al., 2015), leading to variations in stress vulnerability and resilience. It is, therefore, possible that modulation of these systems may enhance resilience in healthcare workers, particularly those exposed to stressful or traumatic situations related to the pandemic. Some of these modulation strategies are discussed in subsequent sections, but others include:

- The prophylactic or early use of antidepressant medications targeting monoamine pathways, particularly in high-risk or frontline staff. Though this approach may not be effective in all cases, there is translational evidence that it may be useful in a subgroup of individuals (Davidson et al., 2005; Nieto-Gonzalez et al., 2015; Han et al., 2017). It is also of interest that some of these drugs have been shown to ameliorate the symptoms of COVID-19 infection, suggesting that they may be particularly useful in healthcare workers who develop COVID-19 (Lenze et al., 2020).
- The experimental use of 5HT_4_ receptor agonists in reducing anxiety and depressive symptoms in healthcare workers exposed to chronic stress (Chen et al., 2020b).
- The use of ketamine, already approved for the acute treatment of depression and suicidal behavior in humans, in subjects at high risk of pandemic-related stress (McGhee et al., 2008; Weinbroum, 2021); the efficacy of this drug appears to depend on intact monoamine pathways (Bowman et al., 2020).

### The Hypothalamic-Pituitary-Adrenal Axis

A substantial body of research has identified the hypothalamic-pituitary adrenal axis as a biological “final common pathway” on which external stress, vulnerability factors, and resilience factors converge. Recent studies have refined the understanding of the complexities involved in HPA axis functioning and regulation (Frodl and O’Keane, 2013). Apart from the established links between HPA axis dysregulation and several common mental disorders (Zorn et al., 2017), recent research has outlined the role of this neuroendocrine pathway in phenomena such as burnout (Kakiashvili et al., 2013; Chow et al., 2018), maladaptive work-related attitudes and practices (Eddy et al., 2018), and responses to discrimination (Busse et al., 2017). These three facets are of particular importance to the situation of healthcare workers during the COVID-19 pandemic, in which chronic workplace stress, high demands and expectations, and stigma related to the risk of infection can all contribute to adverse mental health outcomes (da Silva and Neto, 2020; Dobson et al., 2020; Pappa et al., 2020; Sotgiu and Dobler, 2020; Taylor et al., 2020a). In general, increased workplace stress and a perceived lack of proportionate rewards lead to HPA axis overactivity (Eddy et al., 2018), but in the long run, prolonged stress leading to burnout results in relatively low cortisol levels, despite elevations in corticotrophin-releasing hormone (CRH). These endocrine changes lead to alterations in the methylation of key genes moderating stress response and resilience, such as the glucocorticoid receptor (*NR3C1*) and brain-derived neurotrophic factor (*BDNF*) genes, and to lower levels of BDNF which impair neural plasticity and correlate with the severity of burnout (Bakusic et al., 2017). Relative hypocortisolism is also seen in PTSD, while relative over-activation is seen in depression. Both these conditions are often observed in healthcare workers during the COVID-19 pandemic (Chew et al., 2020).

Recent neurobiological advances provide a number of promising leads for interventions that can positively modulate HPA axis functioning, thereby minimizing the risk of burnout as well as mental disorders in healthcare workers. For example, it has been shown that there is a close link between HRV, a measure of decreased parasympathetic and increased sympathetic nervous system functioning, and regulation of the HPA axis. Reduced HRV is associated with greater dysregulation, and is correlated with impairments in psychological flexibility, social cognition, and resilience to stress (Kemp and Quintana, 2013). Interventions that normalize HRV may lead to improved HPA axis functioning, and protect healthcare workers from a variety of adverse outcomes. A similar relationship has been identified between human circadian rhythms and the HPA axis response to stress; sleep deprivation and frequent changes in sleep-wake schedule can all contribute to dysfunction of this pathway, leading to reduced resilience (Kinlein and Karatsoreos, 2020). This aspect is of particular relevance to healthcare workers involved in frontline or intensive care duties during the COVID-19 pandemic. Finally, the downstream effects of HPA axis dysregulation appear to be related to reduced expression of glucocorticoid receptors (GR) in the hippocampus, a change that can potentially be reversed by pre-treatment with antidepressants in animal models of stress (Han et al., 2017). The latter finding underlines the close links between the HPA axis and monoamine transmission, as discussed in section Monoaminergic Modulation of Stress and Resilience.

Given these complexities, direct pharmacological modulation of the HPA axis may not always yield the expected results, though they may have a role in specific cases. For example, antagonism of CRF receptors would theoretically be expected to enhance resilience; however, CRF-1 antagonists have yielded disappointing results in human subjects to date (Spierling and Zorrilla, 2017). On the other hand, there is promising evidence from controlled clinical trials that administration of low-dose hydrocortisone in the immediate aftermath of trauma could attenuate or even prevent PTSD in inpatients with physical illnesses, perhaps by correcting relative hypocortisolism (Astill Wright et al., 2019). This approach may be useful in healthcare workers who are themselves hospitalized for COVID-19.

In real-world settings, *these findings suggest several promising avenues for building resilience and countering the effects of stress on healthcare workers, through behavioral or pharmacological modulation of the several factors influencing HPA axis functioning*. These interventions can be seen as working chiefly at the level of *plasticity* in Feldman (2018) model Bakusic et al. (2017). Possibilities include:

- The use of techniques that correct reduced HRV, thereby enhancing resilience through HPA axis modulation. These include exercise (Kemp and Quintana, 2013), mindfulness-based interventions (Radmark et al., 2019), yoga-based techniques centered on breathing (Nivethitha et al., 2016), and guided relaxation (Lewis et al., 2015). There is already considerable evidence that such techniques produce significant changes in HPA axis functioning when implemented in workplace settings (Heckenberg et al., 2018).
- Organizational changes aimed at correcting environmental or workplace factors that can contribute to HPA axis dysregulation in the long run. These include due attention to shift work hours to minimize impacts on individual healthcare workers, and efforts to reduce the stigmatization or isolation of those who work with COVID-19 patients and are wrongly viewed as “dangerous” or “infectious” (Taylor et al., 2020a).
- Counseling or self-help techniques aimed, not at general stress reduction, but at correcting factors such as psychological inflexibility and overcommitment to work which are associated with HPA axis dysfunction (Eddy et al., 2018; Guevara and Murdock, 2020) as well as with adverse mental health outcomes in the context of COVID-19 (Landi et al., 2020; Smith et al., 2020). This would regulate this neuroendocrine pathway in a “top-down” manner.
- More speculatively, the use of low-dose steroids in healthcare workers exposed to severe trauma, as this approach has been shown to prevent the development of subsequent PTSD in both translational models and clinical settings (Zohar et al., 2011; Astill Wright et al., 2019).

### Immune-Inflammatory Influences on Stress and Resilience

Over the past three decades, substantial evidence has accumulated on the close links between immune system functioning, responses to stress, and resilience (Breen et al., 2015; Dantzer et al., 2018; Gururajan et al., 2019). Changes in several inflammatory markers, such as elevations in C-reactive protein (CRP), lowered levels of the cytokines interferon-gamma (IFNγ) and tumor necrosis factor-alpha (TNFα), and elevated levels of the chemokines CCL13, CCL20, and CXCL6 have all been associated with an increased risk of PTSD following exposure to traumatic stressors (Eraly et al., 2014; Michopoulos et al., 2020; Zhang et al., 2020a). Conversely, lower levels of interleukin-6 (IL-6) and elevations of the chemokine CX3CL1 have been identified as potential markers of resilience (Imai et al., 2019; Zhang et al., 2020a). In a more general manner, research in animals has shown that exposure to social stress is associated with increase in levels of specific cytokines (IL-2, IL-6, IL-10, IL-17A, IL-22, and TNFα), and these changes are correlated with behavioral responses to stress, and these changes have been associated with reduced neurogenesis and synaptic plasticity (Hodes et al., 2014; Muhie et al., 2017). Given this finding, as well as the intimate reciprocal links between immune and HPA axis functioning in response to experimental models of social stress (Page et al., 2014), it is plausible that alterations in immune function can affect individual resilience at the levels of both *plasticity* and *sociality*. Thus, alterations in immune-inflammatory functioning may represent a mechanism linking both these dimensions. Preliminary evidence in humans has also found evidence of a close link between exposure to social stress and changes in both cortisol and IL-6 levels, which in turn can affect neural plasticity and subsequent responses to adversity (Chen et al., 2017).

As is the case with the HPA axis, there are several promising possibilities for modulating stress-induced changes in immune function and thereby enhancing resilience. From a top-down perspective, reducing social isolation – a particular problem in healthcare workers dealing with the pandemic – has been associated with beneficial changes in peripheral inflammatory markers (Yang et al., 2014; Ahmadian et al., 2020). Similar beneficial effects on immune function have been observed with exercise and dietary changes, as discussed in section Environmental Influences on Resilience: Neurobiological Principles below. From a bottom-up perspective, animal models have shown that immunization with specific substances, such as myelin-related peptides (Lewitus et al., 2008), and certain mycobacterial strains (Reber et al., 2016; Loupy et al., 2021) can attenuate stress-induced anxiety and promote resilience *via* alterations in immune functioning, such as inhibition of stress-related increases in IL-6. The latter finding is of particular significance in the context of the COVID-19 pandemic, as it has been noted that immunization against *Mycobacterium tuberculosis* exerts a potential protective effect against COVID-19 mortality (Li, 2021) and trials of BCG immunization in healthcare workers for this purpose are in progress (Junqueira-Kipnis et al., 2020; Madsen et al., 2020). Stress-induced alterations in immune function are also under epigenetic control and may be amenable to modulation in this manner, as discussed in the next section.

The implications of these findings for healthcare workers are that *it may be possible to identify healthcare workers at a higher risk of adverse outcomes in response to stress by measuring immune-inflammatory markers, and to enhance resilience in staff to stress by direct or indirect modulation of the immune system*. Approaches of possible merit in this regard include:

- Examining the predictive value of immune markers already identified as markers of stress (high CRP, low IFNγ, and TNFα) or resilience (low IL6 and elevated CX3CL1) in prospective studies of healthcare workers.
- Reducing peripheral inflammatory activity by minimizing social isolation and loneliness and fostering mutual and institutional support for healthcare workers.
- Changes in dietary pattern and exercise (discussed in section Environmental Influences on Resilience: Neurobiological Principles below).
- More experimentally, assessing whether BCG immunization is associated with enhanced resilience in data from ongoing clinical trials in healthcare workers, and if this proves to be the case, conducting cautious further trials with this specific outcome in mind.

### Epigenetic Regulation of the Stress Response

Early research into the genetics of resilience focused on candidate genes that were thought to influence the responsiveness of the stress axis, such as monoamine transmitters or HPA axis-related receptors (Jabbi et al., 2007; Derijk and de Kloet, 2008) and then grew to encompass the role of multiple gene-environment interactions, and other genetic variants (Daskalakis et al., 2013). Subsequent studies focused on more downstream molecular mediators of resilience.

A subsequent group of studies focused on molecules that were further downstream in the signal of inter- and intracellular signaling, such as brain-derived neurotrophic factor (BDNF; Notaras and van den Buuse, 2020) as well as genome-wide analyses which have identified novel genes related to psychological resilience, such as doublecortin-like kinase 2 (*DCLK2*) and kelch-like family member 36 (*KLHL36*; Stein et al., 2019). Most of these novel candidates are associated with neuronal integrity and plasticity; thus, these results are in line with a Feldman’s model, in which cellular plasticity is a key mediator of resilience (Feldman, 2018).

However, research into mental disorders such as major depression and PTSD has underlined the key role of gene-environment (GxE) interactions in determining the relationship between genetic variants and mental health outcomes, in what may be termed “two-hit” (genotype x environmental stress) or “three-hit” (genotype x early life adversity x current stress) models (Daskalakis et al., 2013; Zannas and West, 2014). In other words, while genetic variants and childhood adversity may impair resilience, these effects can be buffered by interventions in the “here and now.” A key mechanism underlying this buffering effect is the epigenetic modification of key genes by a variety of environmental factors. These modifications involve chemical changes such as DNA methylation that alter gene transcription and expression without any changes in the actual nucleotide sequence. Environmental stress has been found to exert a marked influence on these processes, both through effects on proteins that regulate methylation, and through effects on “reader” proteins such as methyl-CpG binding protein 2 (*MECP2*) that link DNA methylation to transcriptional activity in key genes, such as the *FKBP5* gene which regulates HPA axis functioning (Reul et al., 2014; Zannas and West, 2014). In fact, it has been suggested that the typical physiological and behavioral responses to stress and trauma in humans are largely caused by epigenetic changes common to many mammals, particularly in genes regulating immune function (Sipahi et al., 2014). Both experimental models of social stress (Nasca et al., 2019) and experiences of stress in real-world settings (Arzate-Mejia et al., 2020) have been associated with demonstrable changes in DNA methylation patterns. In other words, epigenetic mechanisms are another pathway linking the resilience dimensions of *plasticity* and *sociality* in the Feldman’s model.

These changes can, in turn, be potentially reversed through appropriate behavioral, psychological, or even pharmacological interventions, providing a further potential target for interventions aimed at enhancing resilience which can be objectively assessed by measuring changes in DNA methylation (Pape et al., 2018; Gottschalk et al., 2020). Such changes have already been documented for interventions such as meditation (Kaliman, 2019) and psychological therapies (Roberts et al., 2015; Kumsta, 2019), and may prove useful in identifying those who would best profit from such approaches. Beneficial epigenetic changes in GR genes have also been observed in response to psychological interventions in patients with PTSD (Castro-Vale and Carvalho, 2020). It has also been observed that a phytochemical product, dihydrocaffeic acid (DHCA), promotes stress resilience in mice by inhibiting DNA methylation of the interleukin-6 gene (*IL6*; Wang et al., 2018) though such a finding requires replication and testing in human subjects, it represents a promising future intervention strategy for healthcare workers.

In real-world terms, the chief implication of these studies for healthcare workers is that *vulnerability to stress is partly genetically determined, but can be moderated by behavioral and environmental modification*. Potential epigenetics-based approaches in this population could include:

- Assessing changes in methylation of key stress axis genes (*BDNF*, glucocorticoid receptors, and *FKBP5*) in healthcare workers experiencing stress-induced symptoms of anxiety, depression and PTSD, as well as in those making use of workplace stress-reduction programs.
- Provision of early specific trauma-related counseling to frontline healthcare workers, or those showing early signs of traumatic stress while on COVID-19 duty (Castro-Vale and Carvalho, 2020).
- Environmental changes, particularly the provision of emotional and material support (Miller et al., 2015; Shields et al., 2016), which may reverse stress-induced epigenetic changes.
- Experimentally, trials of drugs known to have a positive effect on epigenetic modulation of the HPA axis, immune system, or neuronal plasticity, such as antidepressants (Muñoz-Cobo et al., 2018), antagonists of the corticotropin-releasing factor 1 (CRF) receptor (Pape et al., 2018), and phytochemicals (Wang et al., 2018).

### Neuropeptides

Over the last two decades, a significant body of evidence has accumulated on the key role of neuropeptides in a variety of mental disorders, including anxiety disorders, obsessive-compulsive disorder, PTSD, eating disorders, depression, and alcohol dependence (Bandelow et al., 2017; Harper et al., 2018; Plessow et al., 2018; Shariq et al., 2019). This association may be explained by the fact that neuropeptides co-exist with “classic” neurotransmitters (such as serotonin or dopamine) within neurons, and themselves act as transmitters, neurotrophic factors, and regulators of “classic” neural transmission (Hokfelt et al., 2018). As many of these disorders are triggered or exacerbated by stress, it stands to reason that neuropeptides may prove to be key mediators of resilience at the cellular level. Moreover, neuropeptides are important regulators of social behavior and bonding (Meyer-Lindenberg et al., 2011), making them of direct relevance to the *social* dimension of resilience, particularly in the context of the COVID-19 pandemic where social distancing, quarantine, and reduced social support all exert a negative impact on the mental health of healthcare workers (da Silva and Neto, 2020). Specific neuropeptides have also been strongly correlated with individual variations, religious, and spiritual beliefs (Imamura et al., 2017; Tonnesen et al., 2018), suggesting that – uniquely among the mechanisms discussed thus far – they are also related to the *meaning* dimension of resilience. In other words, from a conceptual viewpoint, neuropeptides are implicated in all three of Feldman’s postulated dimensions of resilience.

Among the various neuropeptides of interest, the most attention has been given to NPY, a 36-amino acid peptide which is widely distributed in the central nervous system. The effects of NPY on resilience are complex: activation of type 1 (Y1) NPY receptors reduces anxiety and mediates resilience, while activation of type 2 (Y2) receptors increase anxiety. On the whole, NPY is considered to have a protective effect against stress, by counteracting the actions of the peptide corticotrophin-releasing hormone (CRH) which activates the “stress axis” (Wu et al., 2013; Reichmann and Holzer, 2016). Administration of NPY reduces submissive and defensive behaviors in male hamsters subjected to social defeat; this effect persisted even after experimental blockade of Y1 receptors, suggesting that other NPY receptor subtypes play an important role in resilience (Lacey et al., 2019). In human subjects, plasma and cerebrospinal fluid levels of NPY correlate negatively with levels of post-traumatic stress in military veterans (Sah et al., 2014), and a functional polymorphism (rs16147) of the *NPY* gene was found to interact with trauma exposure to predict resilience in adults, with the *T* allele conferring a protective role (Gan et al., 2019). Though NPY represents an attractive molecular target for the enhancement of resilience, its effectiveness has not yet been tested in formal pharmacological trials. However, the NPY pathway may be indirectly targeted through modification of gut microbiota or inflammatory activity through the use of probiotics or dietary modification, as gut inflammation has been associated with reduced NPY levels in key brain regions related to stress, such as the hippocampus and amygdala (Holzer et al., 2012).

Besides NPY, a number of neuropeptides have been identified as potential mediators of resilience at the cellular level as well as in terms of influencing adaptive social behavior – in other words, as moderators of *plasticity* and *sociality*. One of the most prominent of these peptides is oxytocin, which appears to exert a regulatory effect on the cortisol response to stress (Li et al., 2019; Winter and Jurek, 2019). In addition, it has been shown to reduce depressive symptoms following loss of a partner in animal models (Bosch and Young, 2018), to reduce brain responses to fear-provoking visual stimuli in human suffering already exposed to trauma (Flanagan et al., 2019), and to potentially enhance the likelihood of engaging in altruistic or pro-social behaviors (Hurlemann, 2017). These effects are all relevant to the COVID-19 pandemic, where healthcare workers often experience interpersonal separation, social isolation, and a heightened exposure to fear-generating cues. Polymorphisms of the oxytocin receptor gene have also been found to influence vulnerability to PTSD (Sippel et al., 2017). These findings suggest that the administration of oxytocin – which is already used as a pharmacological agent in obstetric settings, and as an experimental adjunct to psychological therapies (Domes et al., 2019) – may be effective in increasing resilience and reducing the risk of stress-related mental disorders (Sharma et al., 2020).

Other peptides which have been shown to influence resilience and stress responses, though at a much more preliminary level of evidence, include the endogenous opioid family of enkephalins (Nam et al., 2019), orexin (Staton et al., 2018; Summers et al., 2020), nociceptin (Narendran et al., 2019), somatostatin (Stengel and Tache, 2017), and galanin (Sciolino et al., 2015). These peptides have been shown to enhance resilience in animal models during experimental stress-inducing procedures, and to influence other molecules of key importance in brain resilience, such as BDNF. However, their exact role and significance in humans, and more particularly in the specific situations faced by healthcare workers during the COVID-19 pandemic, requires further investigation.

In real-world settings, the significance of the above findings lies in *the potential to augment resilience to stress, both at the neural and the social levels, through direct (pharmacological) or indirect (diet and exercise) manipulation of brain neuropeptide transmission*. Potential roles for neuropeptide-based interventions in this setting could include:

- The use of lifestyle modifications, such as diet-based interventions or physical exercise, in enhancing the effects of peptides such as NPY and galanin in boosting resilience (Holmes, 2014; Farzi et al., 2015).
- The potential for direct pharmacotherapy using intranasal oxytocin to enhance pro-social behavior and resilience in healthcare workers, either alone or as an adjunct to psychological or behavioral interventions (Koch et al., 2014).
- The possibility of using opioid-based therapies to attenuate the effects of social stress and isolation – “social pain” – in healthcare workers facing specific situations, such as prolonged hours away from home or quarantine, *via* modulation of endogenous opioid receptors. Though a caveat must be raised regarding the possibility of abuse in this context, such treatments may be effective even when used at low doses for short periods of time (Yovell et al., 2016).

### Environmental Influences on Resilience: Neurobiological Principles

The foregoing sections have provided an overview of the myriad biological mechanisms that influence resilience, the relationships between them, and their links to the resilience dimensions of *plasticity*, *sociality*, and *meaning*, particularly in the context of the COVID-19 pandemic. In discussing these, frequent mentions have been made of the influence of environmental factors on the regulation of these processes. It is useful to revisit some of these links from a neurobiological perspective for two reasons. First, certain lifestyle or environmental modifications that have been shown to correlate with specific biological changes, which in turn can be used to objectively assess the effect of such interventions in terms of parameters such as HPA axis functioning, DNA methylation, altered levels of immune markers, or regional brain activity. Second, the knowledge of these correlates could lead to a more purposeful approach to designing and implementing programs to boost resilience in the face of a major crisis, such as the COVID-19 pandemic. Keeping these two objectives in mind, the following are specific domains where experimental knowledge of the biological correlates of resilience and feasible interventions for healthcare workers intersect:

#### Exercise

Regular physical exercise interacts with genetic vulnerability to minimize the risk of post-traumatic stress symptoms, increases HRV, and may positively influence the activity of resilience-promoting neuropeptides. The final common pathway for all these effects may be the expression of the *BDNF* gene in key brain areas involved in resilience, such as the hippocampus and prefrontal cortex, leading to increased local BDNF levels and enhancement of neurogenesis and neural plasticity (Taliaz et al., 2011; Holmes, 2014). In addition, exercise may partially reversing stress-induced changes in immune function (Wang et al., 2020a). Exercise can also exert a beneficial effect on physical health in the context of COVID-19, and the amount of exercise required to achieve these effects – about 15–20 min of walking or other moderate activity per day – is well within the range of what is practical for healthcare workers (Simpson and Katsanis, 2020; Wang et al., 2020a). This aspect is sometimes passed over in standard “stress management” packages for healthcare workers (Chen et al., 2020a), but there are few significant obstacles to its inclusion.

#### Diet

Though firm evidence for a translational link between the gut-brain axis and resilience in humans is lacking (Tooley, 2020), there is evidence that specific nutrients (Toyoda, 2020) or probiotics (Maehata et al., 2019; Westfall et al., 2021) may modulate stress resilience in animal models, most probably by influencing immune function. There is some preliminary evidence to support such an effect in humans (Taylor et al., 2020b), and this approach may be beneficial when planning meals for healthcare workers. Similarly, healthy eating behaviors may be encouraged by instruction and example (Zheng et al., 2020).

#### Housing and Shelter

Animal models have provided a preliminary picture of the complex relationship between housing and stress. In young rodents, but not in adults, single housing is associated with elevated stress compared to group housing. In adult rodents, paired housing evokes a greater stress response than group housing. These effects appear to correlate with the level of expression of the glucocorticoid receptor gene *NR3C1* in the hippocampus (Pan-Vazquez et al., 2015). In addition, the provision of an enriched environment during group housing – which, in animal models, refers to the provision of toys and running wheels – also minimizes the impact of external stressors (van Praag et al., 2000; Huzard et al., 2015). These considerations may be particularly relevant to the living conditions of healthcare workers during the COVID-19 pandemic, where the social isolation caused by individual accommodation (for example, during quarantine) may worsen stress, and the provision of group rest areas and leisure activities may foster resilience.

#### Sleep

Stress has both subjective and objective effects on sleep quantity, quality, and structure, which appear to be mediated by changes in metabotropic glutamate receptor functioning in limbic brain regions (Highland et al., 2019; Sweeten et al., 2021). In addition, sleep deprivation leads to reduced hippocampal neurogenesis and plasticity, potentially impairing resilience to stress (Saletin et al., 2016). In healthcare workers already dealing with long hours or frequent changes in shifts due to the COVID-19 pandemic, these two effects may form a self-reinforcing process, in which sleep deprivation lowers resilience, leading to an increased impact of stress on sleep (Huffmann et al., 2020; Salari et al., 2020). Administrative policies to miminize frequent changes in sleep patterns or prolonged shift work, as well as individual or group behavioral interventions to improve sleep hygiene and sleep-related practices, may prove helpful in minimizing the impact of sleep disruptions on resilience in this population (Elder et al., 2020; Muller et al., 2020; Rajkumar, 2020).

#### Social Support

Evidence from animal research has shown that social involvement, such as the presence of cage mates of the same species during experimental models of stress, significantly increases adaptive behaviors and facilitates fear extinction. These resilience-enhancing effects appear to be associated with changes in the expression of immediate early genes, such as *fos* (Colnaghi et al., 2016). On the other hand, overcrowding, isolation, social defeat, and “social instability” (alternating crowding and isolation) can result in increases in endocrine and behavioral responses to stress in rodent models, an effect which may be partially mediated by the neuropeptide CRH (Beery and Kaufer, 2015) or altered immune functioning (Page et al., 2014). There is evidence that social support is inversely associated with psychological distress in healthcare workers during the COVID-19 pandemic (Alizadeh et al., 2020; Nowicki et al., 2020). In this context, ensuring adequate opportunities for socialization with colleagues, family members, and friends, while adhering to appropriate infection control guidelines, can help in fostering resilience in healthcare workers at the individual and team level, as can attempts to minimize the stigmatization faced by these personnel (Taylor et al., 2020a).

#### Economic and Food Security

Rodent models suggest that scarcity of material resources, such as food, can alter HPA axis functioning and DNA methylation patterns, leading to disturbances in neuroendocrine functioning and social behavior (Perry et al., 2019; Pertille et al., 2020); similar alterations in stress axis functioning in response to poverty or disadvantageous environments have been noted in human children (Finegood et al., 2017) and adults (Sullivan et al., 2019). For a variety of reasons, including work timings, business closures, and stigmatization, healthcare workers may experience insecurity in terms of food, material needs, and income during the COVID-19 pandemic (Cotrin et al., 2020; Larson et al., 2020), particularly in low- and middle-income countries (Onigbinde et al., 2020). Organizational policies that assist healthcare workers in this aspect, both at the workplace and in their homes, may be useful in normalizing endocrine responses to stress and enhancing resilience.

There are several other factors that may be considered in this regard, including the effect of larger-scale social changes, such as those caused by a pandemic, on biomarkers of stress and resilience (Thomaes et al., 2016). However, the purpose of this review is to focus on aspects of environmental change that are supported by translational evidence, and which can be implemented within a reasonable time frame at the institutional or workplace level.

## Conclusion: A Cautionary Tale, Revisited

In the light of the foregoing evidence (see Figure 1), it is now possible to understand what was lacking in the healthcare worker wellness program described in Chen et al. (2020a) and how subsequent modifications substantially improved its acceptability efficacy. Though done unknowingly, many of the changes made in the second wave of this program – the provision of a common rest area and leisure activities, ensuring security in terms of food and other essential supplies, and periodic visits for the purpose of support or counseling – are entirely in line with measures to combat stress and enhance resilience that have proved valuable in experimental models. This is particularly true with reference to the studies summarized section Environmental Influences on Resilience: Neurobiological Principles above, in which exercise, leisure, sleep, and social support can all positively influence the biological and behavioral response to external stress, through mechanisms that are outlined in sections “Monoaminergic Modulation of Stress and Resilience, The Hypothalamic-Pituitary-Adrenal Axis, Immune-Inflammatory Influences on Stress, and Resilience, Epigenetic Regulation of the Stress Response, and Neuropeptides.” However, it is possible to go beyond this. Future programs aimed at building resilience in healthcare workers during and after the COVID-19 pandemic should be multifaceted, and consider the possibility of other neurobiologically-informed approaches to stress modulation, which may include dietary modification, the use of probiotics, mindfulness-based approaches, and even the judicious use of pharmacological agents such as oxytocin, low-dose cortisol, antidepressants, ketamine, or ultra-low-dose opioid agonists in selected cases. This biologically informed approach can also be fruitfully linked with the psychotherapeutic approach advocated by Rosen et al. (2020) – for example, by developing individual or group educational and counseling programs for healthcare workers that focus on specific constructs such as psychological flexibility or the avoidance of overcommitment. Finally, specific biomarkers – involving not only “classical” HPA axis parameters but levels of neuropeptides, genetic polymorphisms, epigenetic alterations, and measures of regional brain activity – could be used both to identify those at high risk of psychological distress, who would benefit from more intensive or sustained interventions, and to obtain objective correlates of the effectiveness of the strategies outlined above. In a context such as the COVID-19 pandemic, it is impossible to avoid stress altogether; moreover, the avoidance of stressors may actually lower resilience (Katz et al., 2019). What is needed is a comprehensive set of approaches that work synergistically to enable healthcare workers to maintain an adaptive level of functioning while minimizing psychological distress, and there is good reason to believe that the methods described in the preceding sections may be valuable additions to this set. Due to space constraints, other neurobiological mediators of resilience, such as the neurotransmitters gamma-amino butyric acid and glutamate (Wagner et al., 2015; Ardi et al., 2019) and the role of microRNAs in influencing the expression of stress-related genes and resilience (Issler et al., 2014), could not be covered in depth; however, they also represent promising future avenues for research and intervention in this field.

In conclusion, there are enough promising leads from both human and animal research – some of which are already being confirmed through field reports, preliminary clinical trials, or both – to suggest that harnessing the potential of the neurobiology of resilience, and placing it at the service of healthcare workers burdened by the COVID-19 pandemic and its attendant stressors, is feasible and may prove to be more efficacious than conventional approaches based on expert opinion. A major challenge for the future will be to integrate these findings into existing services aimed at addressing the mental health needs of healthcare workers, and adapting them to cultural realities as well as to economic and logistic constraints. To ensure the validity of such approaches, both biomarker-based and psychometrically assessed aspects of stress and resilience must be adopted as outcome measures when assessing them in real-world settings.

## Author Contributions

The author developed the concept for this review, carried out the literature search, wrote the paper, and proofread it. This paper represents the author’s original work and has not been submitted for publication elsewhere.

### Conflict of Interest

The authors declare that the research was conducted in the absence of any commercial or financial relationships that could be construed as a potential conflict of interest.
